# Supplementary material for: The causal role of circulating inflammatory markers in osteoporosis: a bidirectional Mendelian randomized study
Source: Front Immunol. 2024 Jul 18;15:1412298. doi: 10.3389/fimmu.2024.1412298 (PMC11291241; doi:10.3389/fimmu.2024.1412298)
Supplement: Supplementary file 1 [file DataSheet_1.zip › supplementary material/OSTEOPOROSIS-CIM/GCST90274805.tsv.gz_forest.pdf]

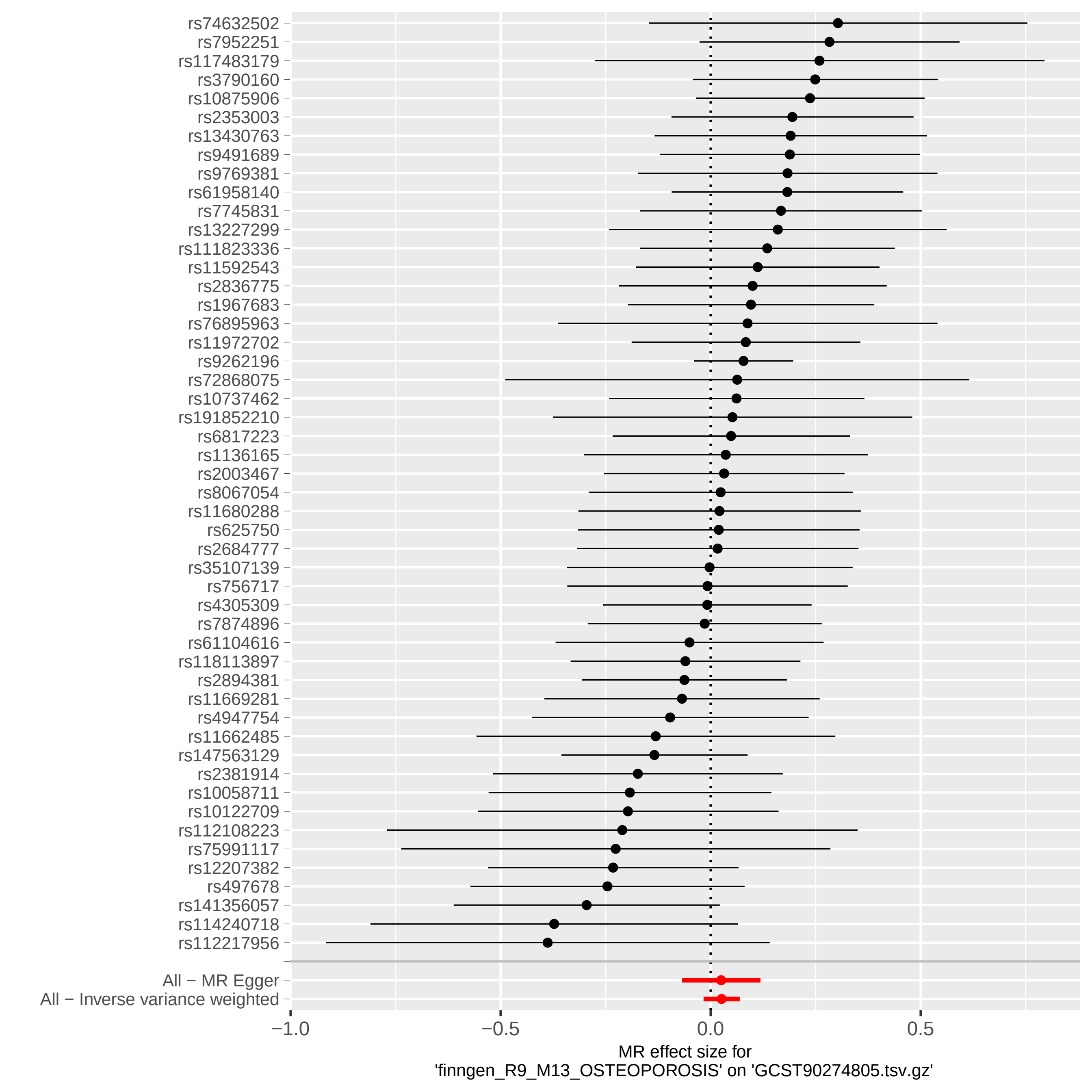

rs74632502
rs7952251
rs117483179
rs3790160
rs10875906
rs2353003
rs13430763
rs9491689
rs9769381
rs61958140
rs7745831
rs13227299
rs111823336
rs11592543
rs2836775
rs1967683
rs76895963
rs11972702
rs9262196
rs72868075
rs10737462
rs191852210
rs6817223
rs1136165
rs2003467
rs8067054
rs11680288
rs625750
rs2684777
rs35107139
rs756717
rs4305309
rs7874896
rs61104616
rs118113897
rs2894381
rs11669281
rs4947754
rs11662485
rs147563129
rs2381914
rs10058711
rs10122709
rs112108223
rs75991117
rs12207382
rs497678
rs141356057
rs114240718
rs112217956
All − MR Egger
All − Inverse variance weighted
−1.0
−0.5
0.0
0.5
MR effect size for
'finngen_R9_M13_OSTEOPOROSIS' on 'GCST90274805.tsv.gz'
